# Reliability of the Cormack-Lehane Classification: A Scoping Review

**DOI:** 10.7759/cureus.81159

**Published:** 2025-03-25

**Authors:** Anoohya Arkala, Maninder Kaur, Joseph Rauscher, Jestin N Carlson, Dhimitri A Nikolla

**Affiliations:** 1 Department of Internal Medicine / Emergency Medicine, Lake Erie College of Osteopathic Medicine, Erie, USA; 2 Department of Emergency Medicine, Allegheny Health Network, Erie, USA

**Keywords:** cormack-lehane classification, endotracheal intubation, inter-rater reliability, kappa, laryngoscopy, modified cormack-lehane classification, scoping review, tracheal intubation

## Abstract

We aimed to find and describe studies estimating the reliability of the Cormack-Lehane and modified Cormack-Lehane classifications, using the kappa statistic (κ). We performed a scoping review searching PubMed as well as Google Scholar and Google.com (gray literature) between October 2024 and January 2025 for published studies without date or language restrictions reporting a κ for Cormack-Lehane grades between at least two raters. We screened 825 records in PubMed and 1,200 in the gray literature of which 15 articles ultimately met our inclusion criteria. Most studies used still images (n=6) and pre-recorded videos (n=8) obtained from a direct (n=5), video (n=5), or fiberoptic (n=4) laryngoscopy (one used both direct and video) performed by clinicians from multiple specialties on patients in the operating room (n=8), simulation (n=2), office (n=3), and prehospital (n=1) settings (one unknown). Studies examined both the Cormack-Lehane classification (n=10) and the modified classification (n=6). Inter-rater reliability ranged from slight to almost perfect, κ from 0.020 to 0.888. The evidence examining the reliability of the Cormack-Lehane and modified Cormack-Lehane classifications is limited with heterogeneous methods and results.

## Introduction and background

Glottic visualization is a critical step in tracheal intubation. Successful laryngoscopy, meaning adequate visualization of the vocal cords, is associated with greater first-attempt success [1,2], and first-attempt success is associated with lower rates of complications [3,4]. First described in 1984 in the context of obstetric anesthesia [5], Cormack-Lehane classification is commonly used to grade the glottic view during intubation.

**Figure 1:**
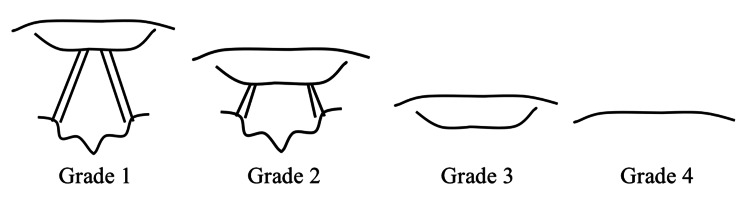
Cormack-Lehane Classification The figure displays the Cormack-Lehane classification grading glottic visualization. Created by: Dhimitri Nikolla

The classification has four grades (1-4), with grade 1 being a full view of the glottis, grade 2 being a partial view, grade 3 being only a view of the epiglottis, and grade 4 being an absent view of the glottis and epiglottis (Figure 1) [5]. The modified Cormack-Lehane classification differentiates Grade 2 views into: 2a being a partial view of the glottis and 2b being only visualization of the arytenoids [6]. Documentation of the Cormack-Lehane grade communicates to other clinicians the potential challenges with laryngoscopy and subsequent intubation [7]. Additionally, the Cormack-Lehane grade is often used to compare peri-intubation interventions, such as laryngoscope and neuromuscular blocking medication choices [1,8,9]. However, the value of the Cormack-Lehane classification has been questioned [10].

The association between the Cormack-Lehane classification and first-attempt success is strong [1,2,11,12]; however, the utility of the classification may be undermined by poor inter-rater reliability (i.e., grades may be inconsistent between raters). Limited data have examined the evidence underpinning its inter-rater reliability. Therefore, we aimed to identify and describe the literature base investigating the reliability of the Cormack-Lehane and modified Cormack-Lehane classifications.

## Review

Materials and methods

Inclusion and Exclusion Criteria

We included all studies performed on human mannequins, cadavers, or patients with vocal cords assessed with the Cormack-Lehane or modified Cormack-Lehane classifications by at least two raters and reported a kappa (κ) statistic for the inter-rater reliability. No exclusions were made on the patient demographic (e.g., age) or participant demographic (e.g., specialty, training level), study setting, publication date, or publication language due to the limited literature and studies published on the reliability of the classifications.

Scoping Search Strategy

We performed structured searches using the PubMed database on January 17, 2025, and gray literature searches using Google Scholar on December 8, 2024 and Google Search on January 15, 2025. We used the following six search terms: ((inter rater reliability) AND (Cormack(Title/Abstract)) AND (Lehane(Title/Abstract))); ((endotracheal intubation(MeSH Terms)) AND (Cormack(Title/Abstract))) AND (Lehane(Title/Abstract)); ((inter-rater) AND (Cormack(Title/Abstract))) AND (Lehane(Title/Abstract)); ((reliable) AND (Cormack(Title/Abstract))) AND (Lehane(Title/Abstract)); ((agreement) AND (Cormack(Title/Abstract))) AND (Lehane(Title/Abstract)); ((kappa) AND (Cormack(Title/Abstract))) AND (Lehane(Title/Abstract)).

Our search was inclusive all years. For the gray literature searches, we screened the first 100 results per search term per source (i.e., Google Scholar and Google search) (Appendix).

Screening

Two independent reviewers (AA, MK) screened the titles and abstracts for the results from the PubMed database and gray literature. After establishing a list of prospective literature, a full-text analysis of each article was performed to determine the inclusion and exclusion criteria. Any discrepancies present were adjusted by a third reviewer (DN).

Data Abstraction and Synthesis

Data were independently abstracted from the full-text articles by two authors (AA, DN) to ensure accuracy. We abstracted variables believed to impact the inter-rater reliability estimates for the Cormack-Lehane grades or affect the generalizability of the study, including view assessed (i.e., still image, recorded video, real-time laryngoscopy), setting (i.e., operating room (OR), office, simulation), participant training/specialty, number of airways assessed in the study, type of laryngoscopy (i.e., direct, video, fiberoptic), number of clinician participants in the study, and classification (Cormack-Lehane or modified Cormack-Lehane). We recorded both inter-rater and intra-rater κ estimates where provided.

We summarized the values for each variable from each study in a table. To better appreciate the heterogeneity between studies, we plotted the inter-rater κ estimates from each study on a scatter plot, indicating the number of clinician participants, type of laryngoscope, and classification. We used R (version 4.4.2, R Foundation for Statistical Computing, Vienna, Austria) to create the visualization. Given the limited number of variables and their objectivity, we did not create a calibrated form for data abstraction. We report our results in alignment with the PRISMA Extension for Scoping Reviews (PRISMA-ScR) checklist [13].

Results

**Figure 2:**
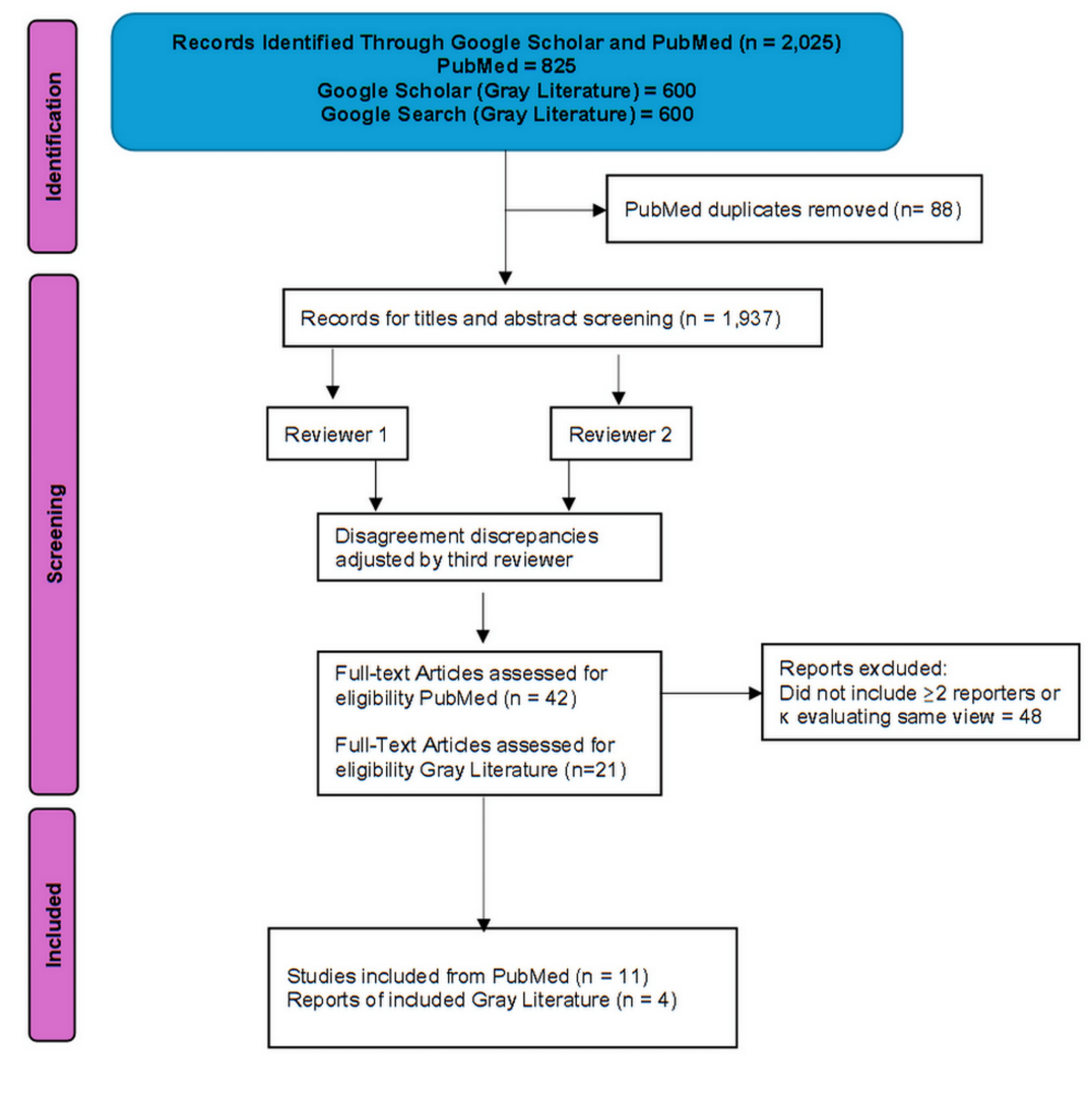
PRISMA diagram of identified studies.

We identified a total of 825 records with 88 duplicates, yielding 737 unique results from six searches performed on the PubMed database. The grey literature search identified four additional records (Figure 2). A total of 15 articles, 11 from PubMed and four from Google Scholar, were included (Table 1) [10,14-27].

**Table 1:** Details of Studies Examining the Reliability of the Cormack-Lehane Classification OR: operating room; OSA: obstructive sleep apnea ^a^The same study with two cohorts where inter-rater reliability was assessed. ^b^Found in the gray literature search. ^c^Modified Cormack-Lehane classification.

Citation	View Assessed	Setting	Clinician Participant Training/Specialty	# of Airways	Type of Laryngoscopy	# of Clinician Participants	Inter-Rater Kappa Statistic (κ)	Intra-Rater Kappa Statistic (κ)
Levitan et al., 1998 [14]	Still Images	OR	Emergency Physicians	25	Direct	4	0.59^c^	0.71^c^
Ochroch et al., 1999 [15]	Still Images	OR	Anesthesiologists	25	Direct	7	0.16	0.83
O’Shea et al., 2005 [16]	Still Images	OR	Paramedics	25	Direct	7	0.22	0.37 to 0.90
Krage et al., 2010 [10]	Real-time laryngoscopy	Simulation	Anesthesiologists and anesthesiology trainees	1 simulator, 4 views	Direct	20	0.35	0.15
O’Loughlin et al., 2017 [17]	Videos	OR	Anesthesiologists and Emergency Physicians	25	Video	74	0.464^c^	0.773^c^
Torre et al., 2018^a^ [18]	Still Images	Office	Otolaryngologist - sleep surgery (expert raters)	90	Fiberoptic	2	0.78 0.87^c^	Not reported
	Still Images	Office	Sleep medicine fellows (novice raters)	60	Fiberoptic	4	0.73 0.65^c^	0.84 0.75^c^
Johnston et al., 2015^b^ [19]	Real-time laryngoscopy for learners and videos of the real-time attempts for the expert raters	Simulation	Medical students, pediatric interns, neonatal fellows, attending neonatologist	1	Direct and Video	58	0.020	Not reported
George et al., 2006 [20]	Video	OR	Anesthesiologists	27	Direct	2	0.61	0.64
Bolzer et al., 2019 [21]	Videos	Office	Otolaryngologists	80	Fiberoptic	2	0.38^c^	Not reported
Van de Perck et al., 2021 [22]	Videos	Office	Otolaryngologists	73	Fiberoptic	2	0.73^c^	Not reported
Sasu et al., 2024 [23]	Videos	Pediatric OR	Anesthetists	904 intubations in 809 patients	Video	2	0.55	Not reported
Naito et al., 2016^b^ [24]	Real-time laryngoscopy for prehospital provider and videos of the real-time attempts for the physician raters	Prehospital	Prehospital nurses, paramedics, and physicians	236	Video	Unknown	0.21	Not reported
Yazicioglu et al., 2021^b^ [25]	Still Images	OR	Anesthesiologists	20	Video	75	0.333	0.743
Lim et al., 2020^b^ [26]	Still Images	OR	Anesthesiologists	14	Fiberoptic	2	0.888^c^ (ramped position) 0.803^c^ (supine position)	Not reported
Garcia-Pintos et al., 2021 [27]	Videos	Unknown	Prehospital providers and physicians	10	Video	60	0.44	Not reported

Six studies utilized still images to evaluate inter-rater reliability [14-16,18,25,26]; three studies utilized real-time laryngoscopy [10,19,24]; eight studies utilized videos [17,19-24,27]. Studies included a variety of participants from different medical specialties and professions. Two articles enrolled emergency medicine physicians [14,17]; seven enrolled anesthesiologists and anesthesiology trainees [10,15,17,20,23,25,26]; three enrolled prehospital providers (paramedics or nurses) [16,24,27]; one enrolled sleep medicine fellows [18]; three studies enrolled otolaryngologists [18,21,22]. Lastly, another study utilized medical students, pediatric interns, neonatal fellows, and attending neonatologists [19].

**Figure 3:**
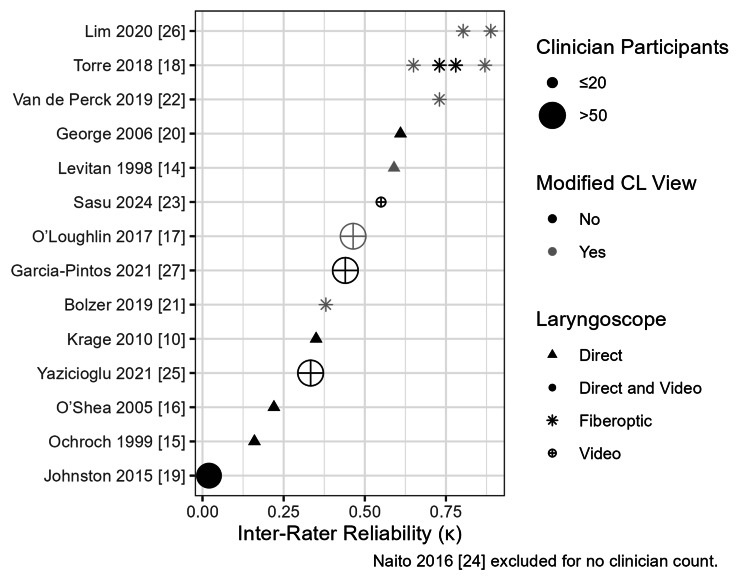
Kappa Estimates by Study Kappa (κ) estimates for inter-rater reliability from each study interacted with the number of clinician participants, use of the modified Cormack-Lehane classification, and laryngoscope type. CL: Cormack-Lehane

Five studies used only direct laryngoscopy [10,14-16,20], five studies used only video laryngoscopy [17,23-25,27], four studies used fiberoptic laryngoscopy [18,21,22,26], and another study used both direct and video laryngoscopy [19]. Six studies examined the inter-rater reliability of the modified Cormack-Lehane classification [14,17,18,21,22,26]. The κ for inter-rater reliability ranged from 0.020 to 0.888 (Table 1, Figure 3).

Discussion

Studies examining the inter-rater reliability of the Cormack-Lehane and modified Cormack-Lehane classifications are limited in number with heterogeneous methods. Among the 11 studies we identified, there was marked variability of κ estimates ranging from 0.020 to 0.888 (slight to almost perfect agreement) [28], which may be explained by the variable study methods. For example, the lowest κ was from a pediatric simulation study using mannequins calculating the Cormack-Lehane view inter-rater reliability by comparing views reported by experts reviewing post-intubation laryngoscopy video recordings to views reported by learners' intubating in real-time with video or direct laryngoscopy [29]. Similarly, the second lowest κ was from a study comparing reported Cormack-Lehane grades from paramedics and nurses intubating in the prehospital setting to physicians reviewing the video laryngoscope videos *post hoc* [24]. The differences in intubation experience and real-time vs. video review bias these κ estimates. Nevertheless, given the limited number of studies, we could not identify any definite trends regarding increased agreement within certain medical specialties or professions (e.g., physician vs. paramedic).

Most studies examined participants' agreement in reviewing images or videos of the vocal cords obtained during laryngoscopy, but varied in the use of different laryngoscopes. Torre et al. used still images obtained by fiberoptic laryngoscopy and observed markedly high inter-rater reliability κ values compared to other studies [18]. This may be due to the quality of images obtained with fiberoptic scopes compared to laryngoscopes. But, these results are likely less generalizable to most intubating clinicians who do not intubate with fiberoptic scopes and likely use direct or video laryngoscopes with blades [30]. However, intubation is a dynamic procedure, and anatomy may or may not be better appreciated by clinicians performing the entire procedure rather than reviewing still images or even videos of only the glottis. For example, in the study by George et al., reliability between Cormack-Lehane views obtained during direct laryngoscopy and from video review with the Airway Cam® (Airway Cam Technologies, Wayne, PA, USA) were substantial but not perfect, 0.63 for intra-observer agreement and 0.70 for inter-observer agreement [20,28]. Therefore, although images and videos of laryngoscopy facilitate more controlled studies, the use of images or videos adds a layer of uncertainty regarding the true agreement two intubators would have intubating the same patient.

We did not identify any studies using real-time laryngoscopy on live patients to assess agreement. Only one study utilized real-time laryngoscopy for all raters, and they used a mannequin with anatomic settings to create expected Cormack-Lehane grades for direct laryngoscopy with a Macintosh blade [10]. Therefore, this study [10], nor others using still images or videos, provide estimates of inter-rater reliability in clinical settings, where anticipation and preparation for difficult intubation are vital.

Furthermore, several of the studies' distributions of Cormack-Lehane grades may not represent current clinical practice, resulting in selection bias. For example, in O'Shea et al. and Levitan et al.'s study, the proportion of Cormack-Lehane grade 3 or 4 views obtained with direct laryngoscopy was 30% (105 of 350 ratings) and 22% (22 of 100 ratings) [14,16]. But, grade 3 or 4 views occur less frequently in more current studies, 11% of prehospital [24], 8-14% of emergency department (ED) or intensive care unit (ICU) [1,2,31,32], and 6-10% of OR intubations [9,11]. This may be due to the more frequent use of video vs. direct laryngoscopy in current practice [1,33]. For example, in two recent randomized control trials comparing direct vs. video laryngoscopy, the prevalence of grade 3 or 4 views was 21% for direct vs. 4% for video in EDs and ICUs and 19% vs. 1% in ORs [9,31]. Nevertheless, the reliability of the Cormack-Lehane classification to distinguish between certain grades may be more important than others. For example, first-attempt success drops more drastically from grades 2 to 3 and 3 to 4 than from grades 1 to 2 with both direct and video laryngoscopy [1,2]. Therefore, a more specific investigation into the reliability of discriminating between certain grades may be more clinically relevant than an overall inter-rater reliability estimate.

The severity of disagreement was not considered in most studies. Only one study utilized weighted κ estimates to account for the severity of disagreement [22]. Since the Cormack-Lehane classification is an ordinal scale, a disagreement of, for example, grade 1 vs. grade 3 between two raters cannot be weighed the same as a disagreement of grade 1 vs. grade 2. Therefore, if most disagreements are within 1 grade of each other, a weighted κ gives more credit for these partial agreements, yielding a higher κ estimate than an unweighted κ [34].

These results do not inform the validity of the Cormack-Lehane view predicting difficult intubation. Cormack-Lehane view is strongly associated with first-attempt success with both direct and video laryngoscopy among experienced and inexperienced clinicians [1,2,11,12]. However, more experienced intubators, the use of video laryngoscopy, and the use of bougies are associated with greater first-attempt success with poor glottic views [1,2]. Therefore, the Cormack-Lehane classification cannot inform whether there will be difficulty with or successful endotracheal tube placement once the best view has been obtained. This is an inherent limitation of the classification, which may be more prevalent with the widespread use of video laryngoscopes [35-37]. For example, the best view of the glottis (i.e., grade 1) may not be optimal when intubating with hyperangulated video laryngoscopy because a restricted view of the glottis may better facilitate endotracheal tube placement than a full view [37]. Some authors have made efforts to develop video laryngoscopy-specific laryngoscopic view classifications, such as the Fremantle Score [33].

Further research is needed to better understand the reliability of the Cormack-Lehane classification. First, given that intubation is a dynamic procedure and anatomy appears different depending on patient positioning, clinician positioning, laryngoscope type, and laryngoscope depth and technique, future work should use real-time laryngoscopy of the same airway with the same device to assess reliability. Furthermore, while technically challenging, future research should investigate the reliability of the Cormack-Lehane view in clinical settings with attention to intubator experience (e.g., resident vs. attending). Also, specific investigations into the reliability of the Cormack-Lehane classification to discriminate between grades 2 and 3 or 3 and 4 views with direct and video laryngoscopy would be more clinically relevant than overall κ estimates. The current literature on the reliability of the Cormack-Lehane view is very limited and cannot be used to draw definitive conclusions on reliability measures (i.e., inter- or intra-).

Limitations

First, given our study purpose was to identify and describe the literature examining the reliability of the Cormack-Lehane and modified Cormack-Lehane classifications, we did not conduct structured critical appraisals of the identified studies, nor structured assessments of the quality of each study. Second, for the PubMed search, we searched the titles and abstracts of studies for our search terms. Therefore, studies with our search terms in the manuscript, but not the title or abstract, may have been missed by our search strategy. Third, we only used one non-gray literature source, PubMed. Fourth, we did not employ a librarian, nor did we preregister a study protocol. Lastly, our search strategy did not include other scoring systems for glottic visualization, such as the percentage of glottic opening (POGO) score [14]. Since the POGO score is more limited in scope (i.e., it only assesses the degree of glottic opening) and it is on a continuous scale (i.e., 0% to 100%) [14], it is not directly comparable to the Cormack-Lehane classification, especially regarding measures of reliability.

## Conclusions

In conclusion, few studies exist examining the reliability of the Cormack-Lehane and modified Cormack-Lehane classifications. Among existing studies, the methods and results are heterogeneous with inter-rater reliability ranging from slight to near perfect agreement. Additional research in the clinical setting is needed to better understand the reliability of the Cormack-Lehane classification.

## Appendices

**Table 2:** Details of Google Search ^a^Counts are not mutually exclusive.

Search Number	Search	# of total results	# of results screened	Total # of records reviewed^a^	# Included after review
1	Cormack AND Lehane Inter Rater Reliability	12,300	100	0	0
2	endotracheal intubation AND Cormack AND Lehane	57,200	100	0	0
3	inter-rater AND Cormack AND Lehane	23,200	100	0	0
4	reliable AND Cormack AND Lehane	28,100	100	0	0
5	agreement AND Cormack AND Lehane	23,700	100	1	0
6	kappa AND Cormack AND Lehane	10,100	100	1	0

**Table 3:** Details of Google Scholar Search ^a^Counts are not mutually exclusive.

Search Number	Search	# of total results	# of results screened	Total # of records reviewed^a^	# Included after review^a^
1	Cormack AND Lehane Inter Rater Reliability	1,500	100	2	1
2	endotracheal intubation AND Cormack Lehane	12,400	100	5	0
3	inter-rater AND Cormack Lehane	175	100	7	1
4	reliable AND Cormack Lehane	3,590	100	3	1
5	agreement AND Cormack Lehane	3,280	100	1	1
6	kappa AND Cormack Lehane	307	100	3	0
